# Comparison of subperiosteal or subgaleal drainage and subdural drainage in patients with chronic subdural hematoma: A systematic review and meta-analysis

**DOI:** 10.1097/MD.0000000000035731

**Published:** 2023-10-27

**Authors:** Ling Song, Kun Zhou, Cheng Wang, Junquan Chen, Bin Feng, Xiaopeng Deng, Xiaolin Du

**Affiliations:** a Department of Pharmacy, The Jinyang Hospital Affiliated to Guizhou Medical University, Guizhou, China; b Department of Neurosurgery, The Jinyang Hospital Affiliated to Guizhou Medical University, Guizhou, China.

**Keywords:** chronic subdural hematoma, drainage, subdural, subgaleal, subperiosteal

## Abstract

**Background:**

Chronic subdural hematoma (CSDH) is a relatively common disease, especially in the elderly, for which there is no clear standard of treatment available. The authors systematically evaluated the efficacy of various surgical procedures for the treatment of chronic subdural hematoma.

**Methods:**

Electronic databases of PubMed, EmBase, Web of Science, Medicine, and the Cochrane Library were searched systematically. Based on the PRISMA template, we finally selected and analyzed 13 eligible papers to evaluate the effect of different drainage methods on CSDH. The primary outcomes were recurrence and clinical outcomes. Secondary outcomes were mortality and postoperative complications and other parameters.

**Results:**

The meta-analysis included 3 randomized controlled trials and 10 retrospective studies (non-randomized controlled trials) involving 3619 patients. The pooled results showed no statistically significant difference between non-subdural drainage (NSD) and subdural drainage (SD) in mortality and complication rates (*P* > 0.05). Additionally, overall pooled results showed that the use of NSD (10.9%) has a lower recurrence rate than the use of SD (11.7%), but the results were not statistically significant (relative risk ratio [RR] = 0.98; 95% confidence interval [CI] = 0.70–1.45; *I*^2^ = 47%; *P* = .92). However, the difference between NSD and SD in postoperative bleeding rate reached statistical significance (RR = 2.39; 95% CI = 1.31–4.36; *I*^2^ = 0 %; *P* = .004). Subgroup analysis showed that SD was associated with similar recurrent CSDH (RR = 0.75; 95% CI = 0.52–1.09; *I*^2^ = 0%; *P* = .14), good recovery (RR = 0.98; 95% CI = 0.93–1.04; *I*^2^ = 0%; *P* = .50), and mortality (RR = 0.98; 95% CI = 0.37–2.57; *I*^2^ = 0%; *P* = .96), compared to NSD.

**Conclusions:**

These results suggest that NSD and SD are equally effective in the treatment of patients with CSDH, with no difference in final clinical characteristics and radiologic outcomes. However, in patients with limited subdural space after evacuation of a hematoma, NSD may be the preferred strategy to avoid iatrogenic brain injury.

## 1. Introduction

Chronic subdural hematoma is a common disease in neurosurgery, it is more common in the elderly population and may reach up to 58.1 and 74 per 100,000 persons for patients 65 years of age or older.^[1]^ Due to the large number of population base, serious population aging, and extended life expectancy, Its incidence trends to raise year by year in the world. The major risk factors for chronic subdural hematoma include age, trauma history, inherited or acquired coagulopathies, antiplatelet therapy, use of anticoagulants, dural vascular abnormalities, and after lumbar puncture.^[2]^ Although chronic subdural hematoma (CSDH) is usually treatable and cured by a neurosurgeons, recurrences have not been eliminated.

The preferred surgical approach remains controversial. Treatment strategies for CSDH lack consistency among neurosurgeons, such as the role of craniotomy, twist drill craniostomy, burr hole, percutaneous subdural tapping and endoscopy, in CSDH amongst various surgeons.^[3]^ Randomized controlled trial (RCT) and meta-analyses have demonstrated that the insertion of a subdural drain after burr-hole drainage of CSDH significantly reduces the rate of recurrence and improves outcome.^[4,5]^ Recently, an increasing number of research have been reported on the outcomes of the use of non-subdural drainage versus subdural drainage after burr-hole craniostomy of symptomatic CSDH. Therefore, the aim of this study was to determine whether non-subdural drainage is safe and effective in preventing recurrence and reducing complications after burr hole evacuation of CSDH, thereby improving functional outcomes.

## 2. Materials and methods

### 2.1. Search strategy

Two independent investigators (L.S. and X.D.) searched PubMed, Web of Science, EmBase, the Cochrane Controlled Trials Register, and Medline from inception to March 2021, for the following keywords:“hematoma, subdural, chronic” (MeSH) OR “CSDH” OR ((“hematoma” OR “hematomas” OR “hematoma” OR “hematomas” OR “hemorrhage” OR “hemorrhage” OR “bleeding” OR “bleed”) AND “subdural” AND “chronic”)) AND (“subperiosteal” OR “subgaleal”) AND (“drain” OR “drains” OR “drainage”). The clearly irrelevant studies were excluded after a step-by-step review of the titles, abstracts, and full text of the reports searched, according to the PRISMA statement. Any disagreement between 2 investigators was resolved by consensus, and if disagreement persisted, a third investigator was required. Contact the authors of the paper if necessary to clarify and provide further information. The search was limited to studies published in English.

### 2.2. Inclusion and exclusion criteria

Inclusion criteria were: diagnosis of CSDH by CT or MRI; treatment methods included subperiosteal or subgaleal drainage and subdural drainage with or without suction; studies with patients 18 years and older; CSDH drainage through burr-hole craniostomy; and RCTs or controlled studies. Studies were excluded if: were non-randomized prospective trials, comparative observational studies, non-comparative observational studies, case reports and population-based registries; drainage performed by endoscopy or craniotomy; studies with patients < 18 years of age; complete data or non-English publication; and meta-analyses, errata, letters, editorials, reviews, and animal experiments.

### 2.3. Data extraction

The data were independently extracted by 2 investigators according to inclusion criteria, including baseline information (authors, year, and type of document) and basic patient characteristics (gender, age, study design, number of cases, postoperative complications, and recurrence). All data were recorded using WPS software. Good functional outcome was defined as a patient’s ability to care for himself or herself and a score of ≤3 on the modified Rankin Scale (mRS) or ≥4 on the Glasgow Outcome Scale. If more than 1 scale was used to assess functional outcomes, we first selected the mRS as the scale and then the Glasgow Outcome Scale. Any disagreement is resolved through discussion and consensus.

### 2.4. Statistical analysis

Raw data were analyzed using Review Manager 5.4 software, and forest plots were generated, with statistical significance at *P* < 0.05. Between studies heterogeneity was assessed by the *I*^2^ statistic and standard chi-square test; heterogeneity was pre-specified at *P* ≤ .10 or *I*^2^ ≥ 50% in this paper. In cases of moderate or low heterogeneity, a fixed-effects model was used for data analysis. Otherwise, existing data were analyzed using a random-effects model. The dichotomous variables were expressed as relative risk ratios with 95% confidence intervals (CI). Continuous variables were evaluated using standardized mean difference. All trials were 2-tailed, and publication bias was assessed using funnel plots.

### 2.5. Ethical approval

These data are based on previous studies, and therefore, for this type of study formal consent is not necessary.

## 3. Results

### 3.1. Study selection

**Figure 1.**
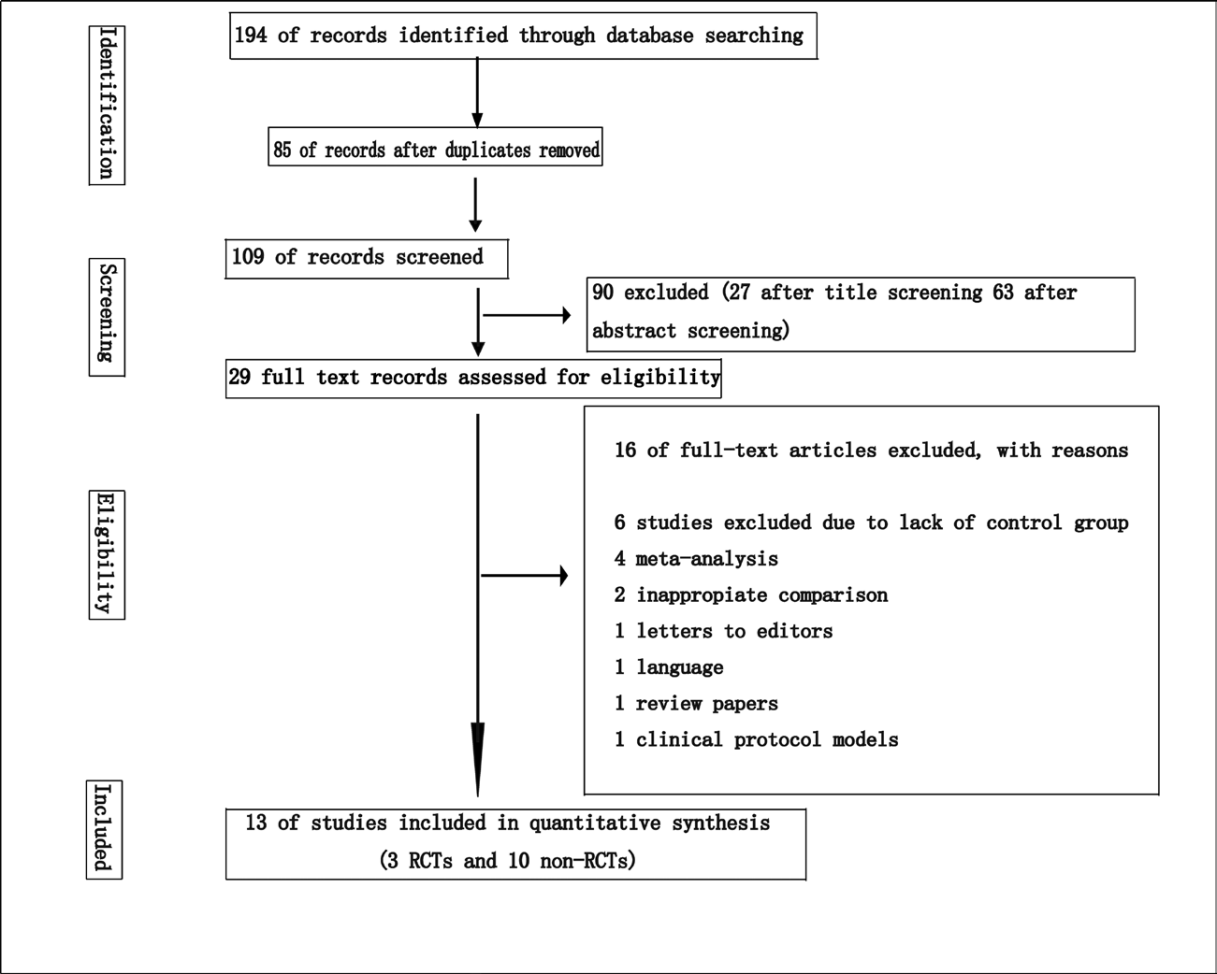
PRISMA flow diagram.

Electronic searches were obtained on 194 of all databases; 85 of these were entered after removal of duplicates. The remaining 109 articles were reviewed for titles and abstracts, and 90 articles were excluded. Full texts of the remaining 29 articles were searched, and 16 articles were excluded for the following reasons:6 studies excluded due to lack of control group; 4 meta-analysis; 2 inappropriate comparison; 1 letters to editors; 1 language; 1 review papers; 1 clinical protocol models. Finally, 13 studies were included in the final analysis, comprising 3 RCTs^[6–8]^ and 10 non-RCTs^[9–18]^ (Fig. 1).

### 3.2. Main characteristics

The primary characteristics of the study included baseline information and patient characteristics (Tables 1 and 2). Each trial described the baseline characteristics of the selected participants, with no significant differences between the non-subdural drainage (NSD) group and the subdural drainage (SD) group. Thirteen studies (3 RCTs and 10 non-RCTs) with a total of 3619 patients (1909 and 1710 patients in the NSD and SD groups, respectively) were included in the meta-analysis of current CSDH therapy.

**Table 1:** Main characteristics.

Study (yr)	Study type	Patients (n) (SD vs NSD)	Mean age (SD vs NSD)	Burr Holes (n)	Follow-up (mo)
Kaliaperumal C (2012)^[6]^	RCT	25 vs 25	73 vs 72	2	6
Soleman J (2019)^[7]^	RCT	100 vs 120	78 vs 81	2	12
Pathoumthong K (2021)^[8]^	RCT	21 vs 21	65	2	6
Chih A (2017)^[10]^	NRCT	30 vs 30	70 vs 68	1	3
Ishfaq A (2017)^[11]^	NRCT	31 vs 31	73 vs 72	1 or 2	within1
Jagminder S (2021)^[17]^	NRCT	35 vs 135	60 vs 60	1 or 2	discharge
Bellut D (2012)^[18]^	Retrospective	65 vs 48	71 vs 77	2	3
Oral S (2015)^[9]^	Retrospective	38 vs 36	66 vs 68	1 or 2	3
Sjavik K (2019)^[12]^	Retrospective	330 vs 764	74 vs 74	1	6
Häni L (2019)^[14]^	Retrospective	214 vs 135	74 vs 73	2	6
Zhang J (2019)^[15]^	Retrospective	329 vs 241	71 vs 70	1 or 2	6
Roberto G (2020)^[16]^	Retrospective	158 vs 80	75 vs 76	1	2
Glancz L (2018)^[13]^	Prospective	533 vs 44	78	1 or 2	2

NSD = non-subdural drainage, RCT = randomized controlled trial, SD = subdural drainage.

**Table 2:** Main characteristics and quality assessment of the selected articles.

Study (yr)	Country	Recurrence (SD vs NSD)	Qualityscore NOS/Robins-1
Kaliaperumal C (2012)^[6]^	Ireland	0 vs 0	–
Soleman J (2019)^[7]^	Switzerland	10 vs 12	–
Pathoumthong K (2021)^[8]^	Thailand	0 vs 2	–
Chih A (2017)^[10]^	Malaysia	1 vs 2	Intermediate
Ishfaq A (2017)^[11]^	Pakistan	3 vs 4	Intermediate
Jagminder S (2021)^[17]^	India	0 vs 1	7
Bellut D (2012)^[18]^	Switzerland	2 vs 1	8
Oral S (2015)^[9]^	Turkey	3 vs 2	7
Sjavik K (2019)^[12]^	Sweden	66 vs 85	8
Häni L (2019)^[14]^	Switzerland	46 vs 37	8
Zhang J (2019)^[15]^	Singapore	43 vs 27	8
Roberto G (2020)^[16]^	Italy	8 vs 10	8
Glancz L (2018)^[13]^	UK	41 vs 4	7

Quality assessment of the retrospective cohort studies using the NOS and of the prospective non-randomized cohort studies using Robins-1.

NOS = Newcastle Ottawa Scale, NSD = non-subdural drainage, RCT = randomized controlled trial, SD = subdural drainage. “-” not applicable.

### 3.3. Quality assessment of the selected articles

**Figure 2.**
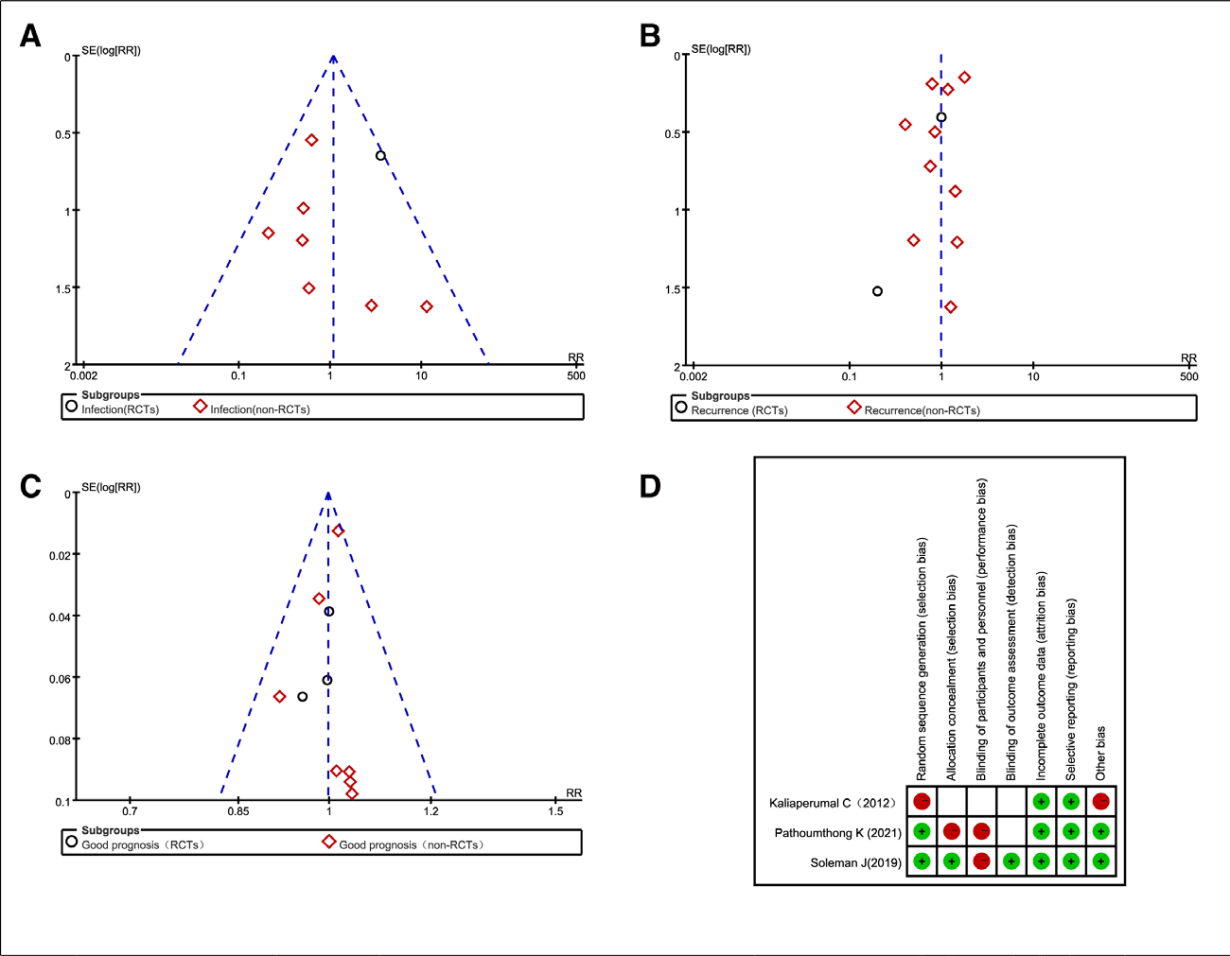
Risk of bias assessment. A symmetrical funnel plots were displayed for intracranial or wound infection (A), recurrent CSDH (B), and good recovery (C). Risk of bias assessment for 3 randomized controlled trials was displayed in (D). CSDH = chronic subdural hematoma.

The quality of RCTs was assessed using Cochrane criteria (Fig. 2D): low risk indicates low-risk bias; high risk indicates high-risk bias; and unspecified risk indicates that reporting does not support adequate or uncertain assessment of information bias. The Newcastle-Ottawa scale was used to assess the quality of retrospective cohort studies and of the prospective non-randomized cohort studies using Robins-1. Table 2 summarizes the risk of bias for all included studies. Additionally, to test whether publication bias was present in the trials included in this meta-analysis, we used a funnel plot (Fig. 2A–C). Although the total number of studies included in this meta-analysis was low, the funnel plots were symmetrically distributed. This result suggests that there is no publication bias.

### 3.4. Pooled results

#### 3.4.1. Intracranial or wound infection in the NSD and SD groups.

**Figure 3.**
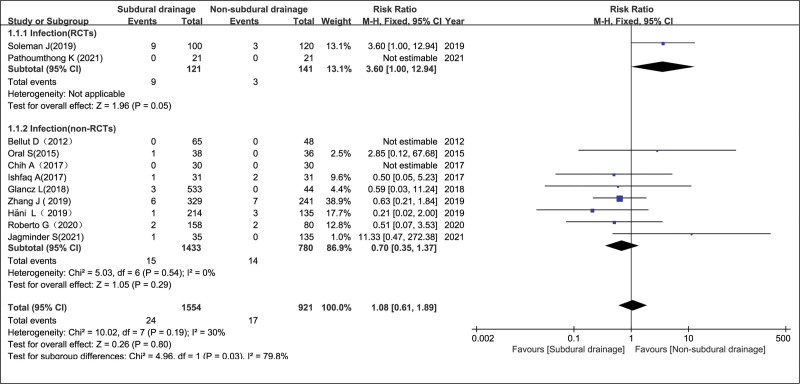
Intracranial or wound infection in the NSD and SD groups. NSD = non-subdural drainage, SD = subdural drainage.

A total of 11 trials^[7–11,13–18]^ (2 RCTs and 9 non-RCTs) comprising 2475 patients (1554 and 921 patients in the Experimental and Control arms, respectively) assessed postoperative intracranial or wound infection, and there was no evidence of statistically significant heterogeneity (non-RCTs, *P* = .29 and *I*^2^ = 0%; RCTs, not available; overall, *P* = .8 and *I*^2^ = 30%). Pooled analysis showed that intracranial and wound infection rates between the NSD and SD groups had no significant differences in the RCT group, non-RCT group and the overall effect, with relative risk ratio (RR) = 3.60 (95% CI = 1.00–12.94, *P* = .05), RR = 0.70 (95% CI = 0.35–1.37, *P* = .29), and RR = 1.08 (95% CI = 0.61–1.89, *P* = .80), respectively, as evaluated with a fixed-effects model. In the forest plot, 2 publications^[6,12]^ were excluded because there were no infections in the NSD and SD groups (Fig. 3). Although the total number of studies included in this meta-analysis was low, the symmetrical distribution of funnel plots was observed, indicating no publication bias (Fig. 2A).

#### 3.4.2. Postoperative bleeding in the NSD and SD groups.

**Figure 4.**
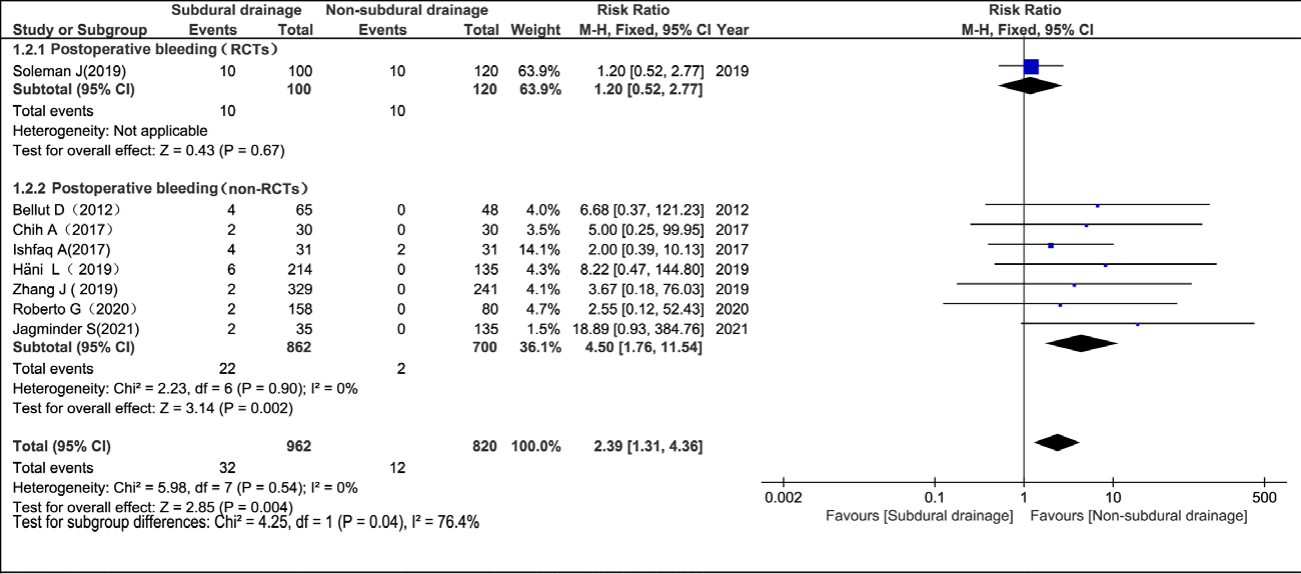
Postoperative bleeding in the NSD and SD groups. NSD = non-subdural drainage, SD = subdural drainage.

In 8 studies^[7,10,11,14–18]^ including 1782 participants, the postoperative bleeding rate was statistically significant in the non-RCT group and overall effect, with RR of 4.50 (95% CI = 1.76–11.54; *P* = .002) and 2.39 (95% CI = 1.31–4.36; *P* = .004), respectively. Meanwhile, there was no significant difference in the RCT group, with RR = 1.20 (95% CI = 0.52–2.77; *P* = .67). There was no evidence of statistically significant heterogeneity (RCTs, not available; non-RCTs, *P* = .90 and *I*^2^ = 0%; overall, *P* = .54 and *I*^2^ = 0%), and a fixed-effects model was used for analysis. In the forest plot, 5 articles were excluded^[6,8,9,12,13]^ because there were no postoperative bleeding cases in the NSD and SD groups (Fig. 4).

#### 3.4.3. Epilepsy in the NSD and SD groups.

**Figure 5.**
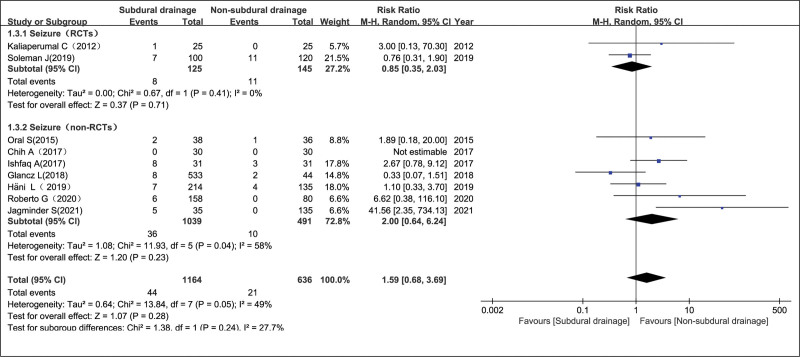
Epilepsy in the NSD and SD groups. NSD = non-subdural drainage, SD = subdural drainage.

Nine studies^[6,7,9–11,13,14,16,17]^ including 1800 participants reported the incidence of postoperative epilepsy, and there was a statistically significant heterogeneity (non-RCTs, *P* = .04 and *I*^2^ = 58%; overall, *P* = .05 and *I*^2^ = 49%), as evaluated with a random-effects model. Pooled analysis showed that epilepsy rates between the NSD and SD groups had no significant differences in the RCT group, non-RCT group and the overall effect, with RR = 0.85 (95% CI = 0.35–2.03, *P* = .71), RR = 2.00 (95% CI = 0.64–6.24, *P* = .23), and RR = 1.59 (95% CI = 0.68–3.69, *P* = .28), respectively (Fig. 5).

#### 3.4.4. Pneumocephalus in the NSD and SD groups.

**Figure 6.**
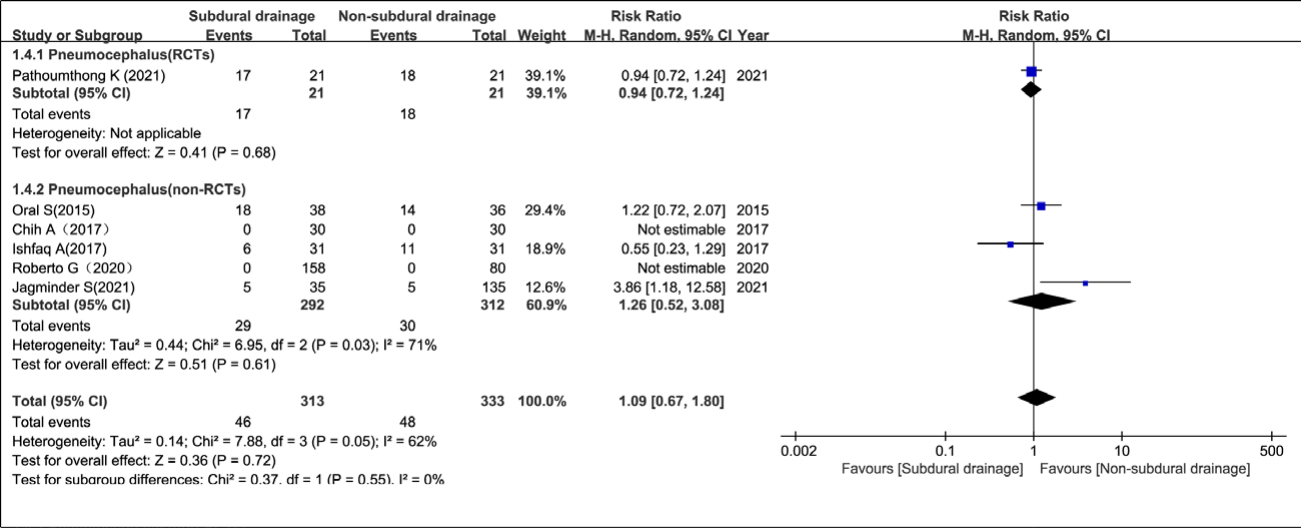
Pneumocephalus in the NSD and SD groups. NSD = non-subdural drainage, SD = subdural drainage.

Six trials^[8–11,16,17]^ consisting of 646 participants reported the rates of pneumocephalus. Pooled analysis showed that epilepsy rates between the NSD and SD groups had no significant differences in the RCT group, non-RCT group and the overall effect, with RR = 0.94 (95% CI = 0.72–1.24, *P* = .68), RR = 1.26 (95% CI = 0.52–3.08, *P* = .61), and RR = 1.09 (95% CI = 0.67–1.80, *P* = .72), respectively, as evaluated using a random-effects model. Measure of consistency according to *I*^2^ was 62% in the overall effect (Fig. 6).

#### 3.4.5. Mortality in the NSD and SD groups.

**Figure 7.**
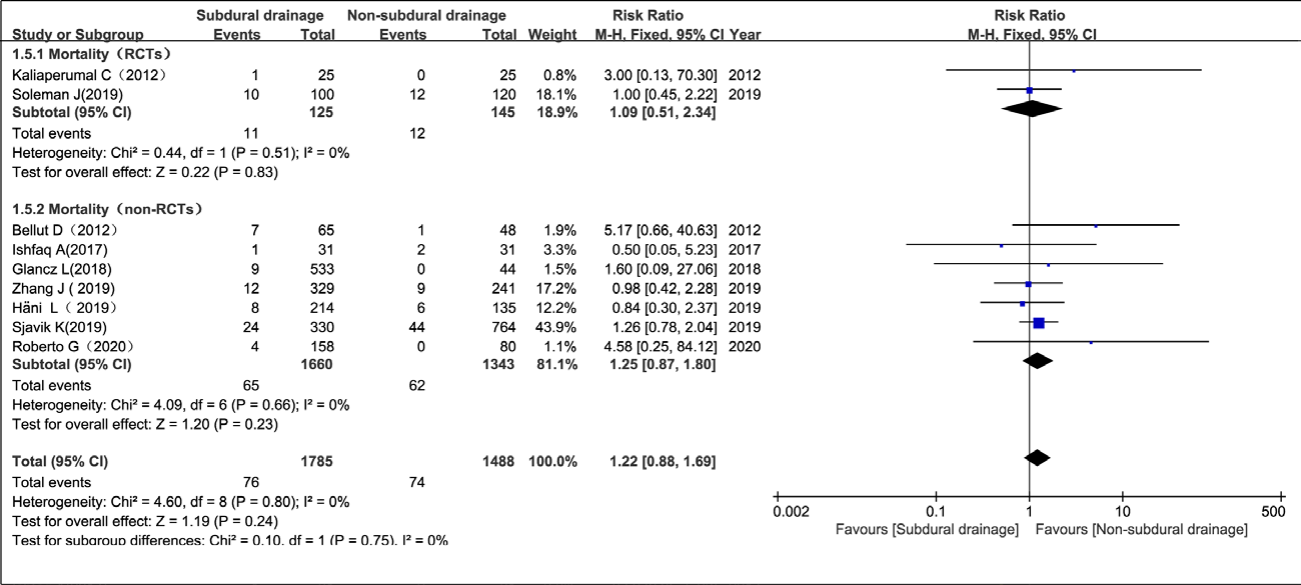
Mortality in the NSD and SD groups. NSD = non-subdural drainage, SD = subdural drainage.

In 9 studies^[6,7,11–16,18]^ evaluating 3273 patients (1785 and 1488 in the SD and NSD groups, respectively), the mortality rates were 42.6% (76/1785) and 49.7% (74/1488) in the SD and NSD groups, respectively. Each study provided information on the effect of SD or NSD on mortality at the end of follow-up. The pooled RRs of death at the end of follow-up using NSD compared to SD for the RCT, non-RCT group and the overall effect showed values of 1.09 (95% CI = 0.51–2.34, *P* = .83), 1.25 (95% CI = 0.87–1.80, *P* = .23), and 1.22 (95% CI = 0.88–1.69, *P* = .24), respectively, indicating that there was no significant difference between the NSD and SD groups. There was no statistically significant evidence of heterogeneity (*P* = .80 and *I*^2^ = 0%), using a fixed-effects model (Fig. 7).

#### 3.4.6. Recurrence in the NSD and SD groups.

**Figure 8.**
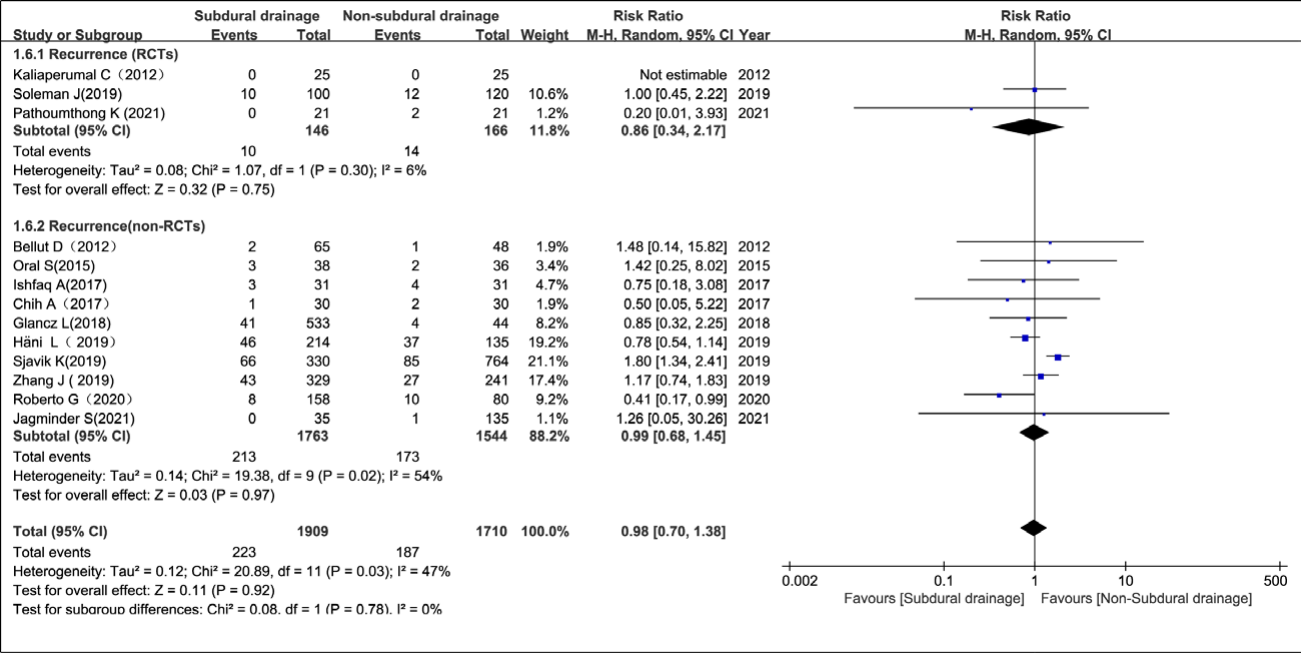
Recurrence in the NSD and SD groups. NSD = non-subdural drainage, SD = subdural drainage.

Thirteen studies^[6–18]^ assessing 3619 patients (1909 and 1710 patients in the SD and NSD arms, respectively) were included in the current meta-analysis of postoperative recurrence. Pooled analysis showed that postoperative recurrence rates between the NSD and SD groups had no significant differences in the non-RCT group, RCT group and the overall effect, with RR = 0.99 (95% CI = 0.68–1.38, *P* = .97), RR = 0.86 (95% CI = 0.34–2.17, *P* = .75), and RR = 0.98 (95% CI = 0.70–1.38, *P* = .92), respectively (Fig. 6). However, there was a significant heterogeneity in the non-RCT group, with *I*^2^ = 54% (*P* = .02), and a random-effects model was used (Fig. 8). This finding is not consistent with previous published literature.^[19]^ Sensitivity analysis also showed no statistical difference between the 2 groups, with the funnel plot visually symmetric (Fig. 2B). There were also no statistical difference in the forest plot of recurrence rate after applying the “leave-one-out” method (data not shown).

#### 3.4.7. Good recovery in the NSD and SD groups.

**Figure 9.**
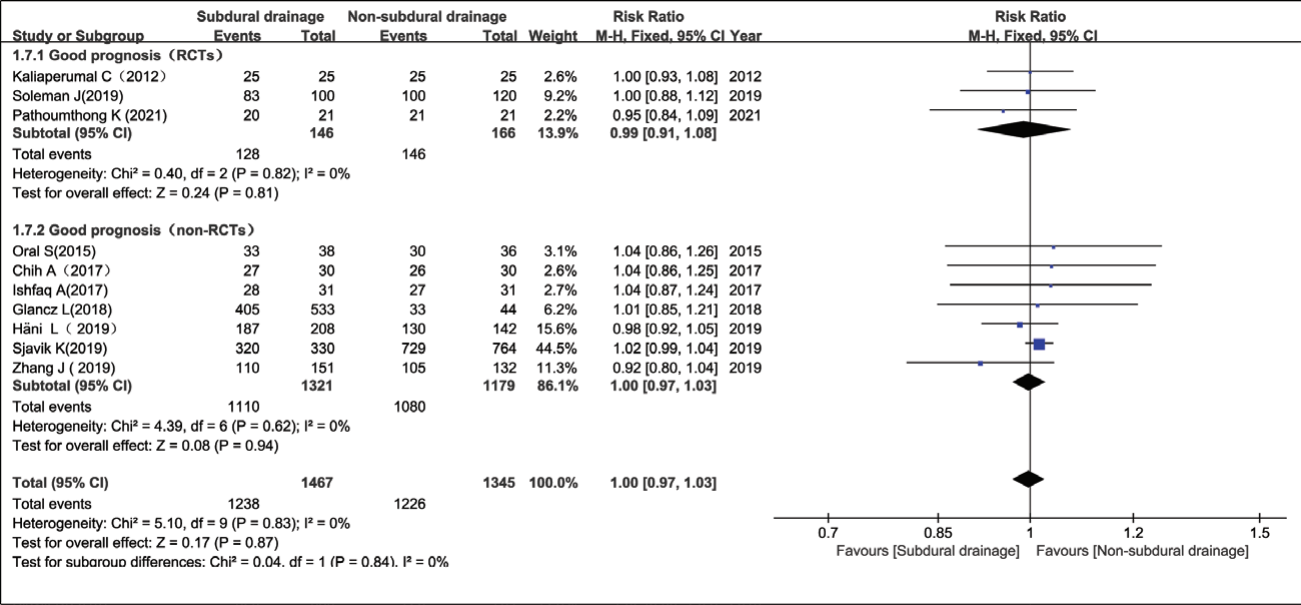
Good recovery in the NSD and SD groups. NSD = non-subdural drainage, SD = subdural drainage.

Ten trials^[6–15]^ assessing 2812 patients (1467 and 1345 patients in the SD and NSD arms, respectively) were included in this study of good recovery. There was no significant differences in good recovery for the RCT, non-RCTs groups and the overall effect, with RR = 0.99 (95% CI = 0.91–1.08, *P* = .81), RR = 1.00 (95% CI = 0.97–1.03, *P* = .94), and RR = 1.00 (95% CI = 0.97–1.03, *P* = .87), respectively. There was no statistically significant evidence of heterogeneity (*P* = .83 and *I*^2^ = 0%), using a fixed-effects model (Fig. 9). Although the total number of studies included in this meta-analysis was low, the symmetric distribution of funnel plots was observed, indicating no publication bias (Fig. 2C).

#### 3.4.8. Subgroup analysis.

**Figure 10.**
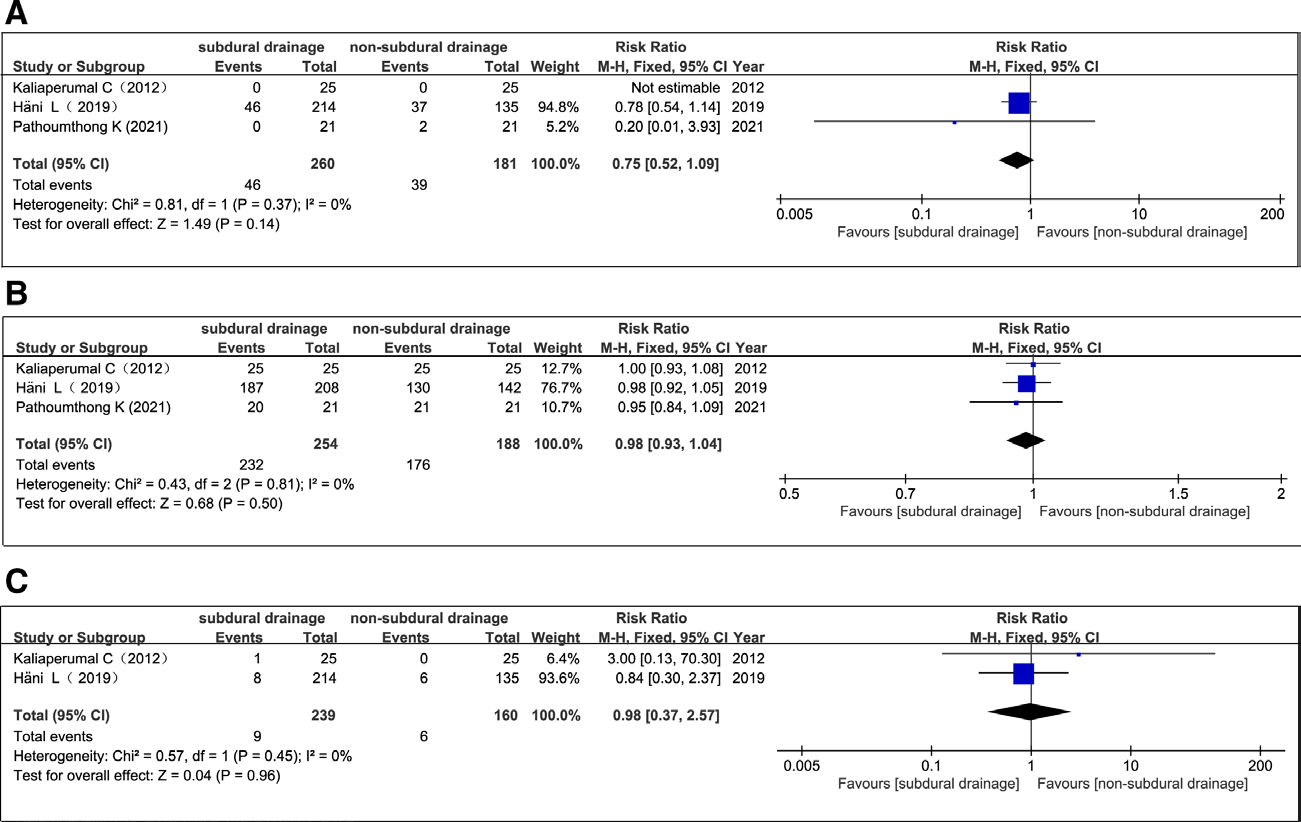
Subgroup analysis for the recurrence (A), good recovery (B), and mortality (C).

Some studies use 2 and others only 1 burr hole, and some studies use active while others use passive drainage, and the follow-up time differ from at discharge to 12 month follow-up. All these features may influence recurrence, outcome and mortality rate. Therefore, we make a more sharply segregation of the used methods, which is to set the follow-up duration at 6 months, with 2 bone holes in all the selected cases. Finally, 3 studies (Kaliaperumal et al,^[6]^ Pathoumthong et al,^[8]^ and Hani et al^[14]^) were included in the study for the analysis, and the results showed that SD was associated with similar recurrent CSDH (RR = 0.75; 95% CI = 0.52–1.09; *I*^2^ = 0%; *P* = .14) (Fig. 10A), good recovery (RR = 0.98; 95% CI = 0.93–1.04; *I*^2^ = 0%; *P* = .50) (Fig. 10B), and mortality (RR = 0.98; 95% CI = 0.37–2.57; *I*^2^ = 0%; *P* = .96) (Fig. 10C), compared to NSD.

## 4. Discussion

CSDH is a relatively common disease, especially in the elderly, for which there is no clear standard of treatment available. Surgical treatment of CSDH has the advantages of preventing herniation, reducing intracranial pressure, improving functional outcome and eliminating recurrence. Despite the widespread use of the CSDH guidelines, accompanying factors such as patient age, Glasgow Coma Scale score at admission, hematoma density, hematoma volume, and middle shift and location often influence neurosurgeon’s decision regarding surgical procedures.^[20]^ Currently, there are many surgical procedures for treating CSDH, including craniotomy, burr hole, twist drill craniostomy and endoscopic surgery, combined with or without subdural drainage. Compared with subdural drainage, non-subdural drainage surgery has no direct contact with brain parenchyma, thus less chances of brain laceration, intracerebral hematoma formation, and seizures, and is highly recommended by many neurosurgeons.^[14]^ However, indiscriminate restrictions on the indications for CSDH based solely on dominance criteria can in some cases increase harm to the patient. Therefore, we performed a systematic review of all available published reports.

In this review, statistical heterogeneity was found between non-subdural drainage and subdural drainage, for recurrence rates, pneumocephalus and epilepsy. A possible reason for heterogeneity is that this study contains multicenter trials that may result from differences in surgical procedures and treatment in many countries or different hospitals in the same country. We performed 2 subgroup analyses of postoperative recurrence and epilepsy, according to country and year of publication, and obtained similar results in this work (data not shown). The jack-knife method and random-effects model were used to analyze pooled data with highly heterogeneous.

There is no international guideline whether a drain should be placed in subdural or non-subdural space after the burr hole. Based on an international survey of practice among neurosurgeons around the world, the inconformity in drain insertion method was reported, Most patients chose subdural drainage (50%) rather than subperiosteal drainage (27%), whereas 23% chose subdural drainage (if available) or subperiosteal drainage.^[20]^ A systematic review published in 2019 noted^[19]^ that non-subdural drainage surgery may have a greater benefit for patients with CSDH compared with subdural drainage. These findings are not consistent with current studies of the clinical effects of subdural drainage. This study provided a different point of opinion in terms of recurrence, but similar rate of mortality, seizures, postoperative bleeding, recovery and infections compared to several previous publications.^[19]^ Additionally, we also assessed the incidence of pneumocephalus and the improvement of postoperative mRS score, in patients administered NSD surgery or SD, adding new contents into this study. To sum up, this may provide a constructive guide for surgeons in selecting the surgical methods for the treatment of CSDH. We consider that NSD can result in better patient prognosis compared with SD when the subdural space is narrow after drainage of the subdural hematoma.

Theoretically, large amounts of residual hematoma are one of the causes of recurrence, poor prognosis and high mortality. It has been concerned recently that the subdural membrane and the overlying dura play a important role in generating the inflammatory exudates that are responsible for hematoma formation and its progression. The presence of high concentrations of fibrin degradation products, plasminogen activator, kallikrein, interleukin-6, platelet-activating factor, fibroblast growth factor and vascular endothelial growth factor in postoperative drainage was associated with a higher rates of hematoma recurrence.^[21,22]^ Postoperative drainage may accelerate the excretion of residual subdural hematoma and brain expansion, and therefore may contribute to early recovery of motor and cognitive function, followed by improved daily activities and self-care.

Incidence of subdural empyema and epilepsy has been reported to be in a range of 0% to 6%, 2% to 19%, respectively, in CSDH patients.^[8]^ In addition, the other common complication is tension symptomatic pneumocephalus. This complication, one of the most common complications after surgery for CSDH, has been reported to range from 16% to 66% in the literature. Gazzeri et al^[23]^ mentioned that subgaleal closed drainage system has a low rate of pneumocephalus and recurrence. We think that drainage tube not in direct contact with the brain tissue and membranes of CSDH may reduce the risk of postoperative seizure and limits the secondary spread of infection to intracranial compartments. However, Patients with preoperative epilepsy still have a high probability of postoperative seizures, and may draw erroneous conclusions. In our study, postoperative infection, pneumocephalus, recurrence and seizures were not statistically significant in the NSD and SD groups.

In addition, different surgical approaches may improve outcomes in patients with CSDH. Based on previous reports and our experience, we believe that a single approach may not be adequate for all patients, and that the operation should be a dialectic choice. To our opinion, choice of surgical technique and type of drainage has to be guided by nerosurgeon preference and intraoperative findings, but, for CSDH patients with limited subdural space after burr-hole drainage of hematoma evacuation, the NSD may be the preferred option to avoid iatrogenic brain injury.

The limitations of this meta-analysis must be pointed out. First, some of the included trials were non-RCTs, and most studies did not report random sequence generation and allocation concealment. Second, there were differences in follow-up duration in these studies. Therefore, more studies are needed to address the complications, good recovery and recurrence, and follow-up for at least 3 months. Third, the relatively limited number of patients included in this study may have affected the results obtained. Additionally, heterogeneity was found in pooled data on recurrence rates, pneumocephalus and epilepsy using random-effects models to more conservatively estimate the overall effect.

## 5. Conclusion

Our findings indicate that NSD and SD are equally effective in treating patients with CSDH, with no difference in final functional outcomes. This study could guide clinicians in the selection of a therapeutic strategy and appropriate patients for NSD surgery in CSDH. However, further RCTs are needed to control for all confounders and confirm this conclusion. At the same time, neurosurgeons should improve their surgical skills to reduce the influence of human factors during surgical procedures.

## Author contributions

**Conceptualization:** Ling Song, Kun Zhou, Xiaolin Du.

**Data curation:** Ling Song, Xiaolin Du.

**Formal analysis:** Cheng Wang, Xiaolin Du.

**Funding acquisition:** Xiaolin Du.

**Investigation:** Junquan Chen, Xiaolin Du.

**Methodology:** Junquan Chen, Bin Feng, Xiaopeng Deng, Xiaolin Du.

**Supervision:** Kun Zhou, Cheng Wang, Xiaolin Du.

**Writing – original draft:** Ling Song, Kun Zhou, Cheng Wang, Xiaolin Du.

**Writing – review & editing:** Ling Song, Kun Zhou, Cheng Wang, Junquan Chen, Bin Feng, Xiaopeng Deng, Xiaolin Du.

## References

[1] Baechli H Nordmann A Bucher HC. Demographics and prevalent risk factors of chronic subdural haematoma: results of a large single-center cohort study. Neurosurg Rev. 2004;27:263–6. 15148652 10.1007/s10143-004-0337-6

[2] Hernandez-Diaz ZM Llibre-Guerra JC Arteche-Prior M. Spontaneous subdural hematoma and behavioral changes due to a dural arteriovenous fistula. A case report and literature review. Behav Sci (Basel). 2019;9:63. 31208005 10.3390/bs9060063 PMC6616428

[3] Yadav YR Parihar V Namdev H. Chronic subdural hematoma. Asian J Neurosurg. 2016;11:330–42. 27695533 10.4103/1793-5482.145102 PMC4974954

[4] Santarius T Kirkpatrick PJ Ganesan D. Use of drains versus no drains after burr-hole evacuation of chronic subdural haematoma: a randomised controlled trial. Lancet. 2009;374:1067–73. 19782872 10.1016/S0140-6736(09)61115-6

[5] Liu W Bakker NA Groen RJ. Chronic subdural hematoma: a systematic review and meta-analysis of surgical procedures. J Neurosurg. 2014;121:665–73. 24995782 10.3171/2014.5.JNS132715

[6] Kaliaperumal C Khalil A Fenton E. A prospective randomised study to compare the utility and outcomes of subdural and subperiosteal drains for the treatment of chronic subdural haematoma. Acta Neurochir (Wien). 2012;154:2083–8; discussion 2088–9. 22932864 10.1007/s00701-012-1483-1

[7] Soleman J Lutz K Schaedelin S. Subperiosteal vs subdural drain after burr-hole drainage of chronic subdural hematoma: a randomized clinical trial (cSDH-Drain-Trial). Neurosurgery. 2019;85:E825–34. 31194877 10.1093/neuros/nyz095

[8] Pathoumthong K Jetjumnong C. Comparative study of subdural drain (SDD) versus sub periosteal drain (SPD) in treating patient with chronic subdural hematoma (CSDH). Surg Neurol Int. 2021;12:421. 34513185 10.25259/SNI_592_2021 PMC8422541

[9] Oral S Borklu RE Kucuk A. Comparison of subgaleal and subdural closed drainage system in the surgical treatment of chronic subdural hematoma. North Clin Istanb. 2015;2:115–21. 28058351 10.14744/nci.2015.06977 PMC5175088

[10] Chih AN Hieng AW Rahman NA. Subperiosteal drainage versus subdural drainage in the management of chronic subdural hematoma (a comparative study). Malays J Med Sci. 2017;24:21–30. 10.21315/mjms2017.24.1.3 PMC5346000 28381926

[11] Ishfaq A. Outcome in chronic subdural hematoma after subdural vs subgaleal drain. J Coll Physicians Surg Pak. 2017;27:419–22. 28818164

[12] Sjavik K Bartek J Jr. Sagberg LM. Assessment of drainage techniques for evacuation of chronic subdural hematoma: a consecutive population-based comparative cohort study. J Neurosurg. 2017;133:1113–9. 10.3171/2016.12.JNS161713 28644099

[13] Glancz LJ Poon MTC Coulter IC.; British Neurosurgical Trainee Research Collaborative (BNTRC). Does drain position and duration influence outcomes in patients undergoing burr-hole evacuation of chronic subdural hematoma? Lessons from a UK Multicenter Prospective Cohort Study. Neurosurgery. 2019;85:486–93. 30169738 10.1093/neuros/nyy366 PMC6761312

[14] Hani L Vulcu S Branca M. Subdural versus subgaleal drainage for chronic subdural hematomas: a post hoc analysis of the TOSCAN trial. J Neurosurg. 2019;133:1147–55. 10.3171/2019.5.JNS19858 31470410

[15] Zhang JJY Wang S Foo ASC. Outcomes of subdural versus subperiosteal drain after burr-hole evacuation of chronic subdural hematoma: a multicenter cohort study. World Neurosurg. 2019;131:e392–401. 31369879 10.1016/j.wneu.2019.07.168

[16] Gazzeri R Laszlo A Faiola A. Clinical investigation of chronic subdural hematoma: relationship between surgical approach, drainage location, use of antithrombotic drugs and postoperative recurrence. Clin Neurol Neurosurg. 2020;191:105705. 32035359 10.1016/j.clineuro.2020.105705

[17] Singh J Sobti S Chaudhary A. Comparative study of subgaleal and subdural closed drain in surgically treated cases of chronic subdural hematoma. Asian J Neurosurg. 2021;16:96–8. 34211874 10.4103/ajns.AJNS_101_20 PMC8202380

[18] Bellut D Woernle CM Burkhardt JK. Subdural drainage versus subperiosteal drainage in burr-hole trepanation for symptomatic chronic subdural hematomas. World Neurosurg. 2012;77:111–8. 22154148 10.1016/j.wneu.2011.05.036

[19] Ding H Liu S Quan X. Subperiosteal versus subdural drain after burr hole drainage for chronic subdural hematomas: a systematic review and meta-analysis. World Neurosurg. 2020;136:90–100. 31927124 10.1016/j.wneu.2019.12.180

[20] Soleman J Kamenova M Lutz K. Drain insertion in chronic subdural hematoma: an international survey of practice. World Neurosurg. 2017;104:528–36. 28461277 10.1016/j.wneu.2017.04.134

[21] Hong HJ Kim YJ Yi HJ. Role of angiogenic growth factors and inflammatory cytokine on recurrence of chronic subdural hematoma. Surg Neurol. 2009;71:161–5; discussion 165–6. 18423527 10.1016/j.surneu.2008.01.023

[22] Kalamatianos T Stavrinou LC Koutsarnakis C. PlGF and sVEGFR-1 in chronic subdural hematoma: implications for hematoma development. J Neurosurg. 2013;118:353–7. 23140147 10.3171/2012.10.JNS12327

[23] Gazzeri R Galarza M Neroni M. Continuous subgaleal suction drainage for the treatment of chronic subdural haematoma. Acta Neurochir (Wien). 2007;149:487–93; discussion 493. 17387427 10.1007/s00701-007-1139-8

